# HSV-1 UL42 and UL47 hijack cellular nucleotide synthetic enzyme CAD to promote lytic replication

**DOI:** 10.1128/jvi.00920-26

**Published:** 2026-09-24

**Authors:** Chao Qin, Xinchi Xie, Taolin Xie, Zhenhao An, Woo-Chang Chung, Pinghui Feng

**Affiliations:** 1 Section of Infection and Immunity, Herman Ostrow School of Dentistry, University of Southern California 43801, Los Angeles, California, USA; 2 Department of Microbial Sciences in Health, Global Center for Pathogen Infection, Center for Experimental Therapeutics, Cleveland Clinic Research 686383, Cleveland, Ohio, USA; University of Virginia, Charlottesville, Virginia, USA

**Keywords:** deamidation, CAD, inflammatory response, pyrimidine synthesis, HSV-1, aerobic glycolysis

## Abstract

Cellular metabolic enzymes are well defined for their roles in metabolism, and their function beyond metabolism is poorly defined. Here, we report that herpes simplex virus 1 (HSV-1) couples evasion of the inflammatory response to metabolic activation to promote lytic replication. Specifically, HSV-1 activates carbamoyl-phosphate synthetase, aspartate transcarbamoylase, and dihydroorotase (CAD), which catalyzes the rate-limiting steps of *de novo* pyrimidine synthesis. Activated CAD not only fuels *de novo* nucleotide synthesis but also deamidates RelA. RelA deamidation suppresses NF-κB activation and the inflammatory response while simultaneously upregulating key glycolytic enzymes to promote aerobic glycolysis. Further affinity purification and functional assays identified HSV-1 UL42 and UL47 that interact with and activate CAD, recapitulating the phenotypes of RelA deamidation. Collectively, our work uncovers a role for a metabolic enzyme in coupling immune evasion to metabolic activation through its protein deamidase activity during HSV-1 infection, highlighting a potential therapeutic target.

**IMPORTANCE:**

Host immune defense and cellular metabolism are two fundamental processes that shape viral pathogenesis. Here, we revealed that herpes simplex virus 1 (HSV-1) activates a nucleotide synthetic enzyme not only to support nucleotide synthesis but also to exploit its unconventional activity for immune evasion and metabolic reprogramming, highlighting a potential therapeutic target.

## INTRODUCTION

As a ubiquitous pathogen in humans, herpes simplex virus type 1 (HSV-1) establishes latent infection in trigeminal ganglia (TG) sensory neurons and undergoes lytic replication in various locations, resulting in various mucocutaneous diseases, such as herpes labialis, genital herpes, herpetic whitlow, and keratitis. It also causes serious encephalitis in a small portion of infected individuals (1–3). To persist within an immune-competent host, HSV-1 has evolved numerous intricate strategies to manipulate, evade, and exploit the host immune response (4, 5). For example, HSV-1 ICP27 interacts with TANK-binding kinase 1 (TBK1) and stimulator of interferon genes (STING) to impede interferon (IFN) regulatory factor 3 (IRF3) activation, thereby reducing IFN production (6). The tegument protein of HSV-1, UL37, deamidates retinoic acid-inducible gene-I (RIG-I) and cyclic GMP-AMP synthase (cGAS) to abolish their activation and subsequent antiviral immune response (7, 8). HSV-1 encodes ICP0, an E3 ligase, to induce the degradation of a number of host antiviral factors in a proteasome-dependent manner, including interferon-γ-inducible protein 16 (IFI16) (9). HSV-1 tegument protein VP22 was reported to inhibit AIM2-dependent inflammasome activation and IL-1β secretion through interacting with AIM2 and preventing its oligomerization (10). A kinase encoded by HSV-1, US3, inhibits the expression of IFN-stimulated genes (ISGs) through degrading Bcl-2-associated transcription factor 1 (Bclaf1), a novel regulator of IFN signaling (11). These studies elucidate the key roles of host immune pathways and viral evasion thereof.

UL42 and UL47 are critical HSV-1 factors that govern viral replication. UL42 functions as an essential DNA polymerase processivity factor, binding directly to the catalytic subunit UL30 to act as a sliding clamp that ensures efficient viral DNA synthesis without premature dissociation (12, 13). Additionally, UL42 inhibits the host antiviral response by muting type I interferon production and NF-kB activation (14, 15). UL47 (also known as VP13/14) is an abundant structural tegument protein that dynamically shuttles between the nucleus and cytoplasm. It binds viral RNA to modulate gene expression and facilitates capsid wrapping and viral egress during assembly (16–18). These two proteins highlight the virus’s sophisticated dual reliance on precise enzymatic regulation and multifunctional structural components to successfully infect and replicate within host cells.

Host immune defense and cellular metabolism are two fundamental processes that shape the pathogenesis of viral infection (19). Like other viruses, HSV-1 depends on cellular machinery for its lytic replication. To meet the voracious demands of energy and macromolecules, HSV-1 activates key metabolic enzymes to provide metabolites for its replication. For example, HSV-1 was reported to activate glycolysis, likely through increasing the expression of phosphofructokinase-1 (PFK-1) and subsequently upregulating its enzymatic activity (20). HSV-1 infection also elevates the biosynthesis of nucleotides, particularly the *de novo* synthesis of pyrimidines (21). How HSV-1 hijacks cellular metabolic pathways to regulate immune response is poorly understood. Gaining insight into how exactly metabolic enzymes and immune response are coordinated during viral infection will advance our understanding of the fundamental mechanisms of HSV-1 replication, which, in turn, provides new perspectives for antiviral therapy.

Protein deamidation, a process to convert glutamine and asparagine residues to glutamate and aspartate, respectively, was originally thought of as a spontaneous reaction and associated with protein “aging” (22). Until recently, a small subset of protein deamidations was reported to critically regulate protein functions. Several studies demonstrated that bacteria secreted effector proteins deamidate and inactivate key innate immune signaling molecules, thus dismantling host immune defense (23, 24). Interestingly, viral and cellular deamidases play vital roles in dampening the innate immune response via targeting key players (7, 8, 25–28). Here, we report that HSV-1 activates *de novo* pyrimidine synthesis through carbamoyl-phosphate synthetase 2, aspartate transcarbamylase, and dihydroorotase (CAD). Activated CAD not only fueled nucleotide synthesis but also blunted the inflammatory response and promoted aerobic glycolysis via deamidating RelA. HSV-1 UL42 and UL47 activate CAD to promote pyrimidine synthesis and evade the inflammatory immune response. Thus, HSV-1 effectively couples immune evasion to metabolic evasion via CAD-mediated RelA deamidation, thereby promoting viral replication.

## RESULTS

### HSV-1 activates CAD to promote *de novo* pyrimidine synthesis

**Fig 1:**
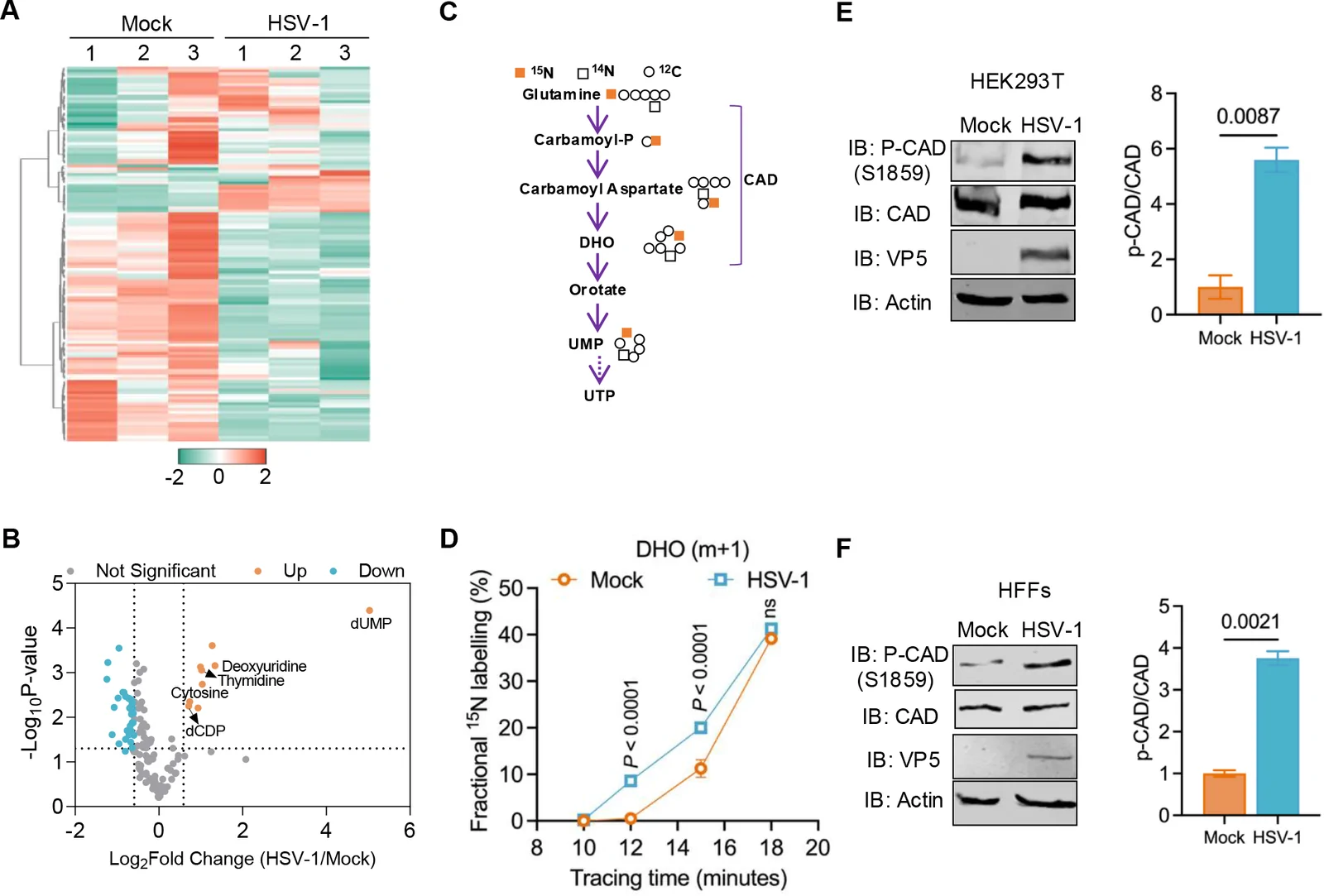
HSV-1 activates CAD to promote *de novo* pyrimidine synthesis. (**A**) Total detected metabolite change in mock or HSV-1-infected HFFs (MOI = 5, 10 h). (**B**) A volcano plot showing significantly changed metabolites in mock or HSV-1-infected HFFs (MOI = 5, 10 h). (**C**) Diagram of the isotope labeling of selected metabolites of pyrimidine metabolism using [amide-^15^N]glutamine. (**D**) Fraction of the heavy isotopologues of dihydroorotate (DHO) in mock or HSV-1-infected MEFs (MOI = 5, 6 h) traced with [amide-^15^N]glutamine for indicated time points. (**E and F**) Immunoblots of whole-cell lysates (WCLs) of mock or HSV-1-infected HEK293T (**E**) and HFFs (**F**) (MOI = 5, 10 h). The ratio of phosphorylated CAD to total CAD was assessed by intensity quantification. Data are presented as means ± SD of biological triplicates (**B and D**) and are representative of two independent experiments (**E and F**). Statistical analysis was performed by the two-way ANOVA test (**D**) or unpaired, two-tailed Student’s *t*-test (**E and F**).

To examine HSV-1-induced metabolic reprogramming, we collected metabolites from mock- and HSV-1-infected human foreskin fibroblasts (HFFs) and analyzed them by liquid chromatography-mass spectrometry (LC-MS). HSV-1 infection significantly altered the overall landscape of metabolites, with some increased or decreased, while others remained unchanged (Fig. 1A). A volcano plot of significantly altered metabolites revealed that most metabolites upregulated by HSV-1 infection were pyrimidines, including dUMP, deoxyuridine, thymidine, cytosine, and dCDP (Fig. 1B). This finding is consistent with previous studies (21). *De novo* synthesis is essential for maintaining the pyrimidine pool, and CAD is the rate-limiting enzyme of the *de novo* pyrimidine biosynthesis pathway (Fig. 1C). To assess the metabolic activity of CAD, we then performed tracing experiments using isotope-labeled [amide-^15^N]glutamine. HSV-1 infection in MEFs significantly increased the levels of heavy isotopologues of dihydroorotate (DHO), the immediate product of CAD, at 12 and 15 min after tracing, which quickly saturated at 18 min, while having no effect on the total pool size of DHO (Fig. 1D and Fig. S1A). HSV-1 infection also significantly increased the heavy isotopologues of DHO and other downstream products, including UMP and UTP in HFFs (Fig. S1B through D). These results indicate that HSV-1 infection increases the CAD-mediated *de novo* pyrimidine synthesis. The activity of CAD in synthesizing pyrimidines is increased by phosphorylation of serine 1859 (S1859) mediated by the ribosomal protein S6 kinase 1 (S6K1) (29, 30). We then analyzed the S1859 phosphorylation of CAD in cells with or without HSV-1 infection. Immunoblotting analysis indicated that HSV-1 infection increased CAD S1859 phosphorylation four- to six-fold compared to mock-infected controls (Fig. 1E and F). Together, these results demonstrate that HSV-1 activates CAD to promote *de novo* pyrimidine synthesis.

### A pyrimidine-independent role of CAD in promoting HSV-1 lytic replication

**Fig 2:**
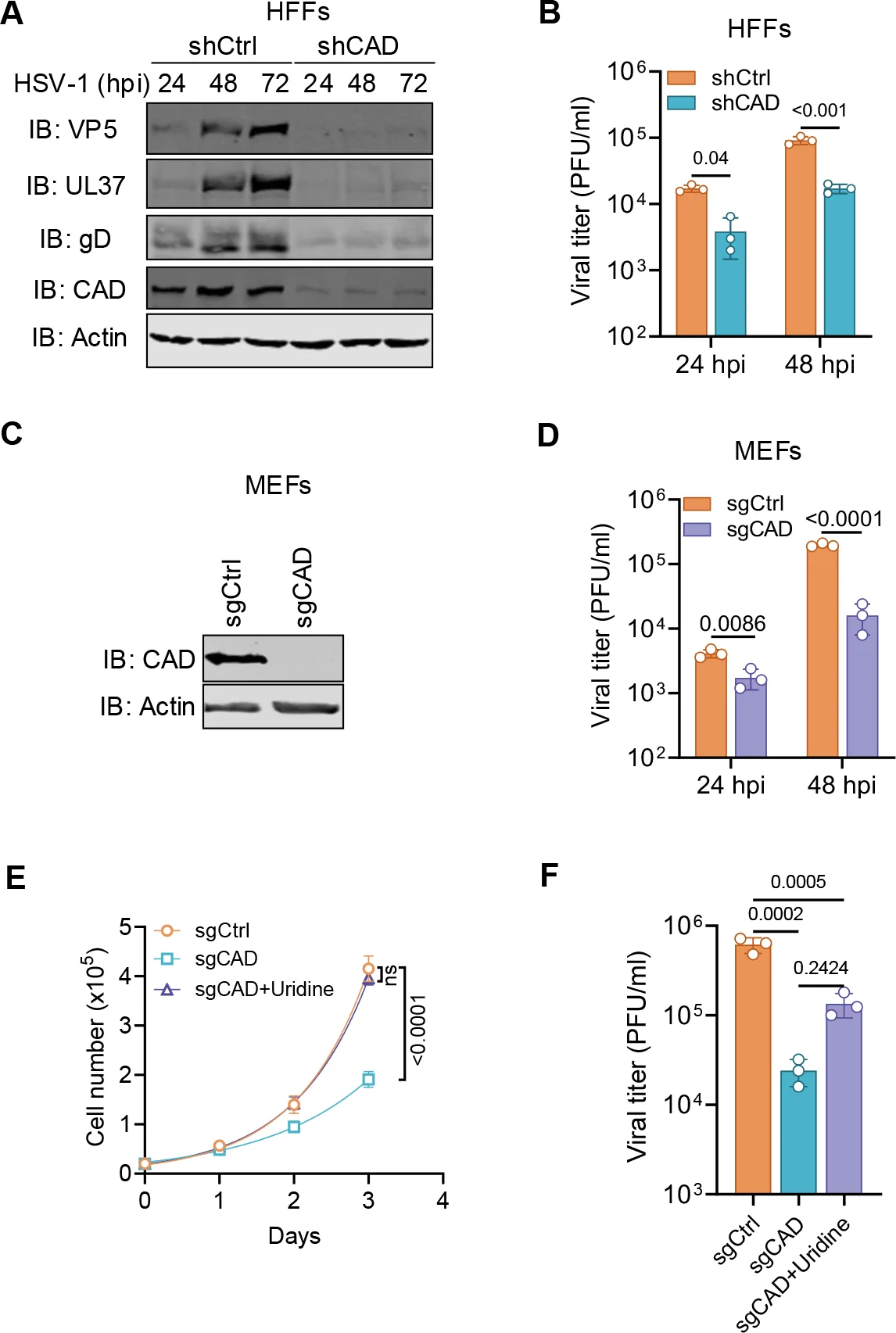
A pyrimidine-independent role of CAD in promoting HSV-1 lytic replication. (**A**) Immunoblots of whole-cell lysates (WCLs) of HSV-1-infected control or CAD-depleted HFFs (MOI = 0.1) at indicated time points. (**B**) Viral titer in HSV-1-infected control or CAD-depleted HFFs (MOI = 0.1) at indicated time points. (**C**) Immunoblots of whole-cell lysates (WCLs) of control or CAD-deleted MEFs. (**D**) Viral titer in HSV-1-infected control or CAD-deleted MEFs (MOI = 0.1) at indicated time points. (**E**) Cell proliferation of control, CAD-deleted MEFs, or CAD-deleted MEFs supplemented with 100 μM uridine. (**F**) Viral titer in HSV-1-infected control, CAD-deleted MEFs, or CAD-deleted MEFs supplemented with 100 μM uridine (MOI = 0.1, 48 hpi). Data are presented as means ± SD of biological triplicates (**B, D, E, and F**) and are representative of three independent experiments (**A and C**). Statistical analysis was performed by the two-way ANOVA test (**B and D**) or one-way ANOVA test (**E and F**).

We then assessed the role of CAD in HSV-1 replication. CAD depletion by short hairpin RNA (shRNA) in HFFs markedly reduced viral protein expression, including major capsid protein VP5, tegument protein UL37, and glycoprotein D (gD) (Fig. 2A). Compared with control cells, CAD-depleted cells exhibited a significant 4-fold reduction in viral titer at 24 h post-infection (hpi) and a 10-fold reduction at 48 hpi (Fig. 2B). We also deleted CAD in mouse embryonic fibroblasts (MEFs) using CRISPR/Cas9 and determined HSV-1 lytic replication (Fig. 2C). Deletion of CAD in MEFs resulted in a greater than 10-fold reduction in HSV-1 titer following infection at a multiplicity of infection (MOI) of 0.1 (Fig. 2D). To determine whether impaired viral replication in CAD-deleted cells stems from reduced pyrimidine pools, we supplemented CAD-deleted MEFs with uridine. In mock-infected MEFs, although CAD deletion reduced cell proliferation by more than three folds, uridine supplementation fully restored it, indicating that the pyrimidine pools were effectively restored in CAD-deleted cells by uridine (Fig. 2E). Notably, uridine supplementation only partially restored HSV-1 titer reduced by CAD deletion, suggesting a pyrimidine-independent mechanism by which CAD promotes HSV-1 lytic replication (Fig. 2F). Together, these data demonstrate an essential role of CAD in HSV-1 replication that is not solely attributed to its function in pyrimidine biosynthesis.

### HSV-1 promotes CAD-mediated RelA deamidation to diminish inflammatory response

**Fig 3:**
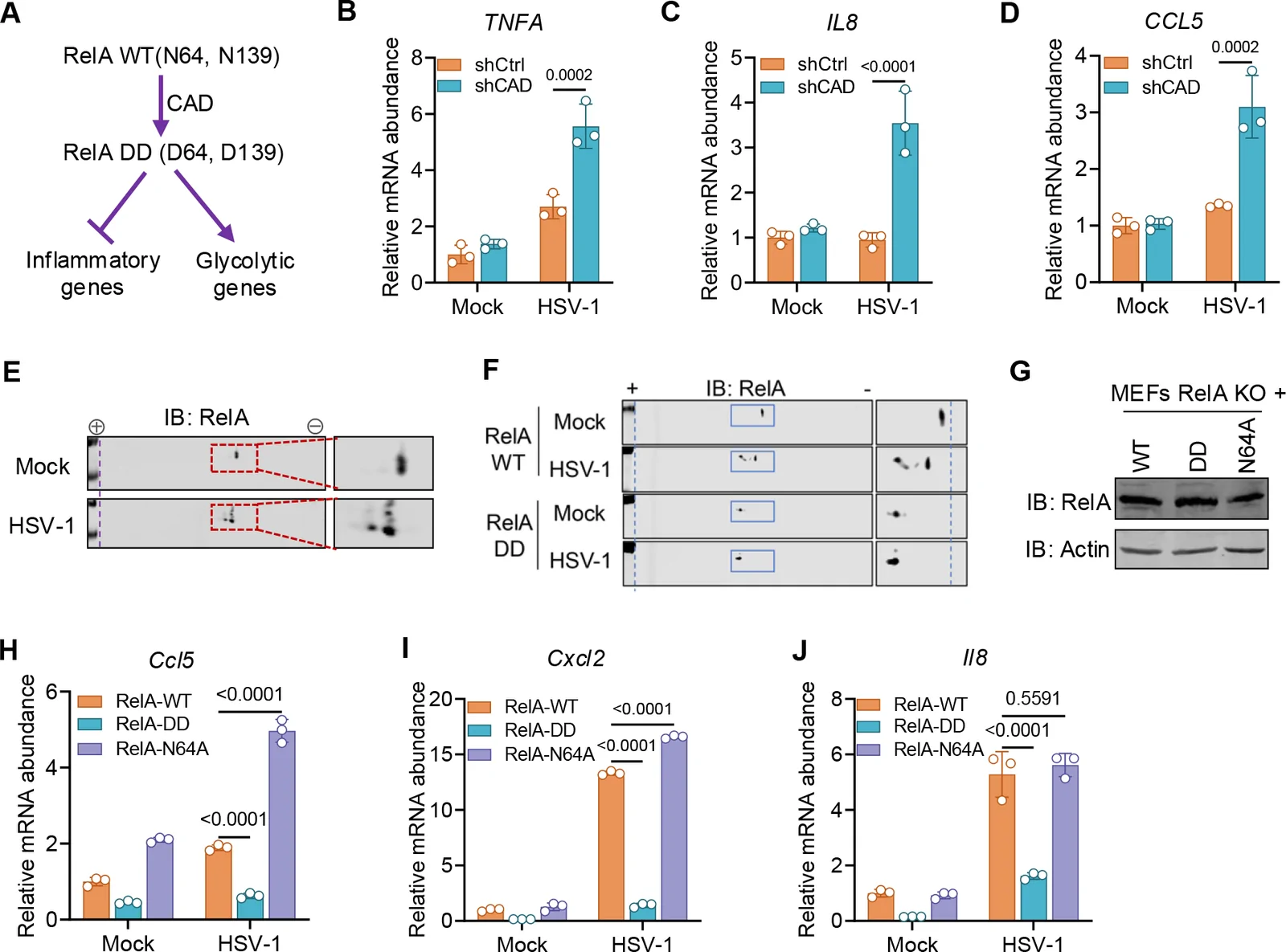
HSV-1 promotes CAD-mediated RelA deamidation to block inflammatory response. (**A**) Diagram of function of CAD-mediated RelA deamidation. (**B–D**) Expression of inflammatory genes in control or CAD-depleted HFFs analyzed by real-time PCR (MOI = 3, 6 h). (**E**) Immunoblots of whole-cell lysates (WCLs) of mock or HSV-1-infected HFFs (MOI = 5, 10 h) analyzed by two-dimensional gel electrophoresis (2-DGE). (**F**) Immunoblots of WCLs of mock or HSV-1-infected HCT116 cells expressing wild-type or deamidated (DD) RelA (MOI = 5, 10 h) analyzed by 2-DGE. (**G**) Immunoblots of WCLs of RelA-KO MEFs reconstituted with wild-type, deamidated (DD), or deamidation-resistant (N64A) RelA. (**H–J**) Expression of inflammatory genes in mock or HSV-1-infected reconstituted MEFs analyzed by real-time PCR (MOI = 3, 6 h). Data are presented as means ± SD of biological triplicates (**B–D and H–J**) and are representative of three independent experiments (**E–G**). Statistical analysis was performed by the two-way ANOVA test (**B–D and H–J**).

We previously reported that CAD deamidates RelA to modulate inflammatory response (26). RelA deamidation at N64/139 by CAD blocks the expression of NF-κB-responsive genes and promotes the expression of glycolytic genes (Fig. 3A). We then tested whether the pyrimidine-independent role of CAD in promoting HSV-1 replication is due to CAD-mediated RelA deamidation. We first depleted CAD and examined antiviral gene expression upon HSV-1 infection. Real-time PCR analysis revealed that CAD depletion led to a two- to four-fold increase in the expression of antiviral inflammatory genes in HSV-1-infected HFFs (Fig. 3B through D). We then examined whether HSV-1 infection induces RelA deamidation in HFFs. Indeed, HSV-1 infection shifted RelA toward the positive end of the gel strip, as analyzed by two-dimensional gel electrophoresis (2-DGE), consistent with the outcome of deamidation (Fig. 3E). Moreover, HSV-1 infection did not significantly shift RelA in cells expressing RelA-DD (26), confirming that HSV-1 infection primarily increases RelA deamidation at N64 and N139 (Fig. 3F). To study the role of CAD-mediated RelA deamidation in inflammatory response, we reconstituted RelA-knockout (RelA-KO) MEFs with wild-type RelA, deamidated RelA-DD (containing N64D and N139D), or deamidation-resistant RelA-N64A (Fig. 3G) and analyzed the expression of NF-κB-responsive genes upon HSV-1 infection. Compared with wild-type cells, RelA-DD decreased the expression of *Ccl5*, *Cxcl2*, and *Il8* by 3- to 10-fold during HSV-1 infection, whereas RelA-N64A enhanced that of *Ccl5* and *Cxcl2* (Fig. 3H through J). These results demonstrate that HSV-1 promotes CAD-mediated RelA deamidation to dampen antiviral inflammatory gene expression.

### HSV-1 promotes CAD-mediated RelA deamidation to upregulate glycolysis

**Fig 4:**
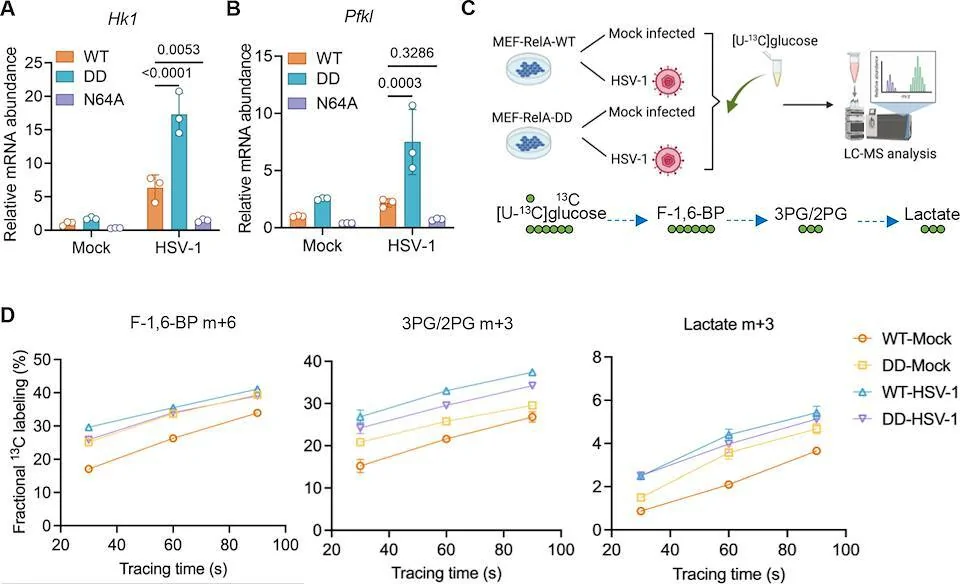
HSV-1 promotes CAD-mediated RelA deamidation to upregulate glycolysis. (**A and B**) Expression of glycolytic genes in mock- and HSV-1-infected reconstituted MEFs analyzed by real-time PCR (MOI = 3, 6 h). (**C**) Diagram of the isotope labeling of selected metabolites of glycolysis using [U-^13^C]glucose. (**D**) Fraction of the heavy isotopologues of indicated metabolites in RelA-KO MEFs reconstituted with wild-type or deamidated (DD) RelA traced with [U-^13^C]glucose (HSV-1 infection: MOI = 5, 6 h). Data are presented as means ± SD of biological triplicates (**A, B, and D**). Statistical analysis was performed by the two-way ANOVA test (**A and B**).

RelA deamidation can induce the expression of key glycolytic genes, although diminishing inflammatory gene expression (26). We next determined whether RelA deamidation increases the expression of these metabolic enzymes during HSV-1 infection. Indeed, the expression of glycolytic genes, such as *Hk1* and *Pfkl*, was significantly higher in RelA-DD MEFs than that in wild-type MEFs infected with or without HSV-1. Conversely, their expression was significantly reduced by three-fold in HSV-1-infected RelA-N64A MEFs (Fig. 4A and B). To further explore the role of RelA deamidation in the glycolytic pathway during HSV-1 infection, we traced mock- and HSV-1-infected MEFs with [U-^13^C]glucose and analyzed the heavy isotopologues of key glycolytic intermediates (Fig. 4C). LC-MS analysis revealed that deamidated RelA increased the labeling of the glycolytic intermediates, including fructose-1,6-bisphosphate (F-1,6-BP), 3-phosphoglycerate (3PG)/2-phosphoglycerate (2PG), and lactate, in mock-infected MEFs, while it had no significant effect on the pool size of these metabolites (Fig. 4D and Fig. S2). Moreover, HSV-1 infection enhanced glycolytic intermediate fractional labeling in WT MEFs; it exerted a negligible effect on that in RelA-DD cells (Fig. 4D). We speculate that the elevated baseline metabolic state of mock-infected RelA-DD MEFs is already high, making it hard to further increase by HSV-1 infection. Notably, although mRNA levels of glycolytic genes were higher in RelA-DD cells than those in WT MEFs under both mock- and HSV-1-infected conditions, RelA-DD expression failed to further elevate glycolysis upon HSV-1 infection. This phenotype may reflect the necessity of dynamic deamidation, rather than statically and fully deamidated RelA, commanded by HSV-1 during lytic replication. In essence, the combination of wild-type and deamidated RelA, including the ratio between wild-type and deamidated RelA and the site-specific deamidation of N64 and N139, may impose distinct metabolic and other biological outputs. Thus, CAD-mediated RelA deamidation activates glycolysis to support HSV-1 lytic replication.

### CAD-mediated RelA deamidation promotes HSV-1 replication

**Fig 5:**
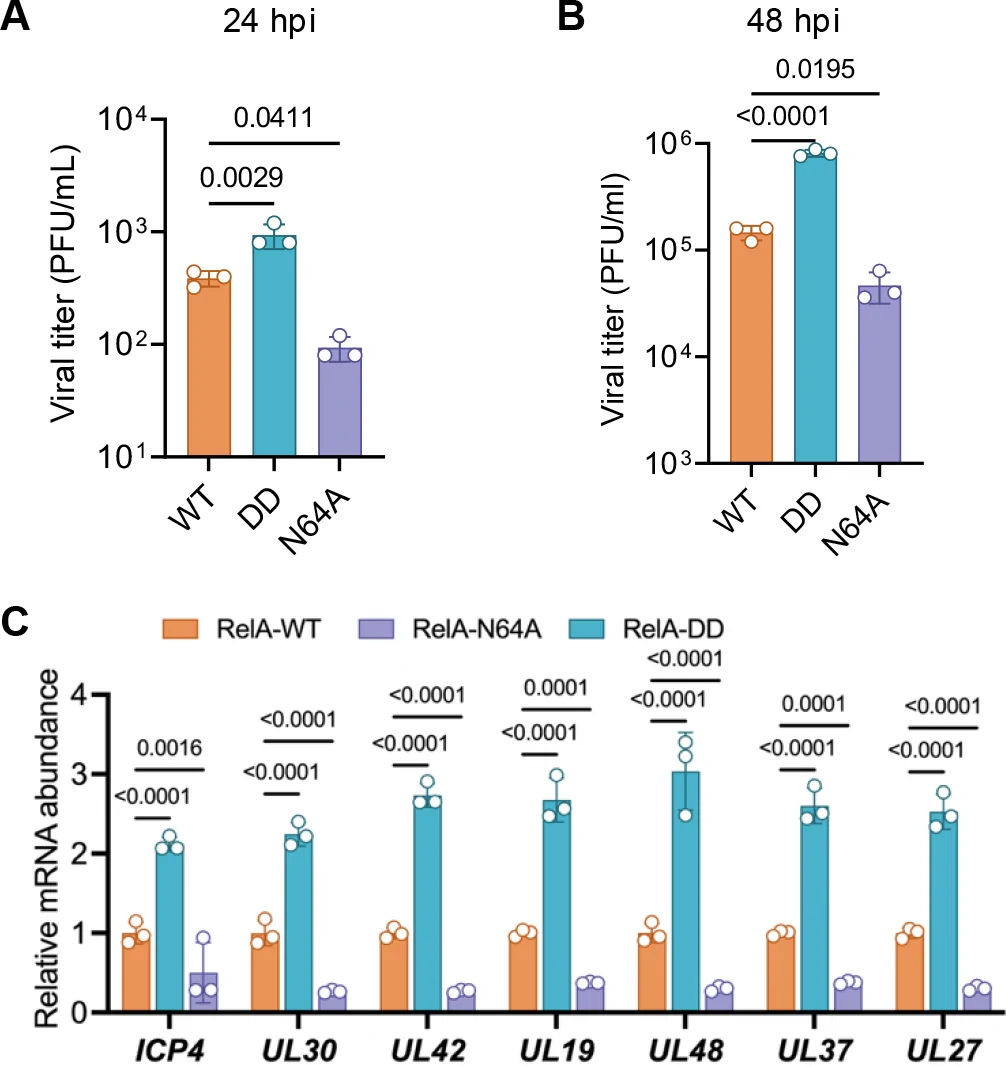
CAD-mediated RelA deamidation promotes HSV-1 replication. (**A–C**) Viral titer at indicated hours (**A and B**) and viral gene expression at 48 hpi (**C**) in RelA-KO MEFs reconstituted with wild-type, deamidated (DD), or deamidation-resistant (N64A) RelA (MOI = 0.1). Data are presented as means ± SD of biological triplicates (**A–C**). Statistical analysis was performed by the one-way ANOVA test (**A and B**) or two-way ANOVA test (**C**).

We next investigated the role of CAD-mediated RelA deamidation in HSV-1 lytic replication. In reconstituted MEFs expressing wild-type RelA, deamidated RelA (RelA-DD), or the deamidation-resistant mutant RelA-N64A, HSV-1 viral titer was increased in RelA-DD MEFs by 2.5-fold at 24 hpi and 8-fold at 48 hpi compared to wild-type MEFs. Conversely, titer in RelA-N64A MEFs was reduced by more than threefold at both time points (Fig. 5A and B). Consistently, real-time PCR analysis of viral gene expression showed that RelA-DD increased, whereas RelA-N64A decreased, mRNA levels of viral genes by two- to three-fold (Fig. 5C). Together, these results indicate that CAD-mediated RelA deamidation promotes HSV-1 lytic replication.

### HSV-1 UL42 and UL47 activate CAD

**Fig 6:**
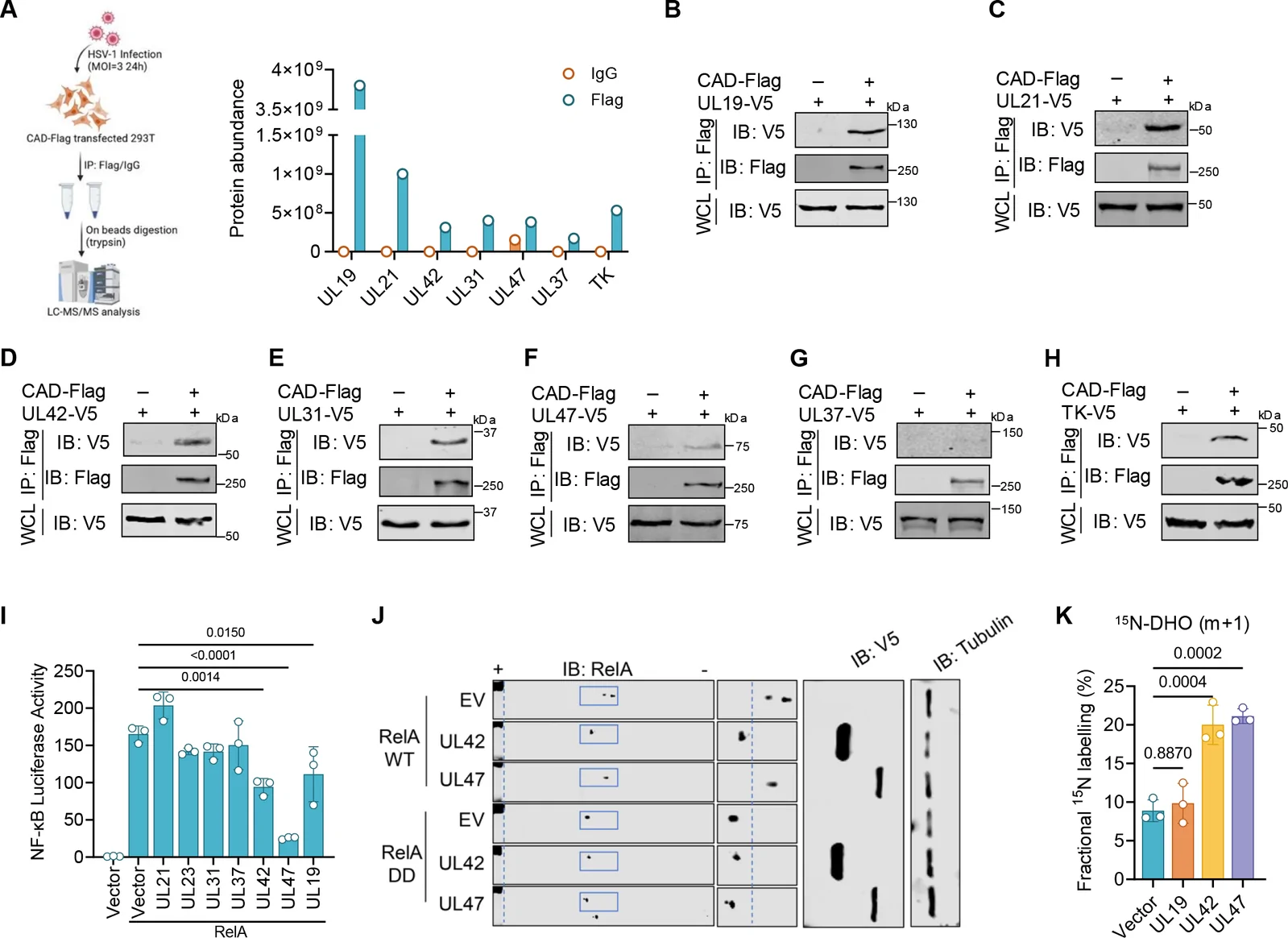
HSV-1 UL42 and UL47 activate CAD. (**A**) CAD-binding proteins in HSV-1-infected cells stably expressing CAD-Flag were identified by affinity chromatography and tandem mass spectrometry. (**B–H**) Immunoblots of precipitated proteins and whole-cell lysates (WCLs) from HEK293T cells expressing CAD-Flag and V5-tagged indicated viral proteins. (**I**) HEK293T cells were transfected with plasmids expressing RelA or HSV-1 proteins, and NF-κB luciferase reporter plasmid mixture. Luciferase activity was determined at 24 h after transfection. (**J**) Immunoblots of whole-cell lysates (WCLs) of HCT116 cells expressing wild-type or deamidated (DD) RelA, transfected with empty vector or plasmids expressing UL42 or UL47 analyzed by two-dimensional gel electrophoresis (2-DGE). (**K**) Fraction of the heavy isotopologues of DHO in 293T cells transfected with empty vector or plasmids expressing UL19, UL42 or UL47 traced with [amide-^15^N]glutamine for 15 min. Data are presented as means ± SD of biological triplicates (**I and K**) and are representative of three independent experiments (**B–H and J**). Statistical analysis was performed by the one-way ANOVA test (**I and K**).

To investigate the viral mechanism underlying HSV-1-mediated CAD activation, we sought to identify viral proteins that interact with CAD. HEK293T cells were transfected with FLAG-tagged CAD and subsequently infected with HSV-1. Proteins were immunoprecipitated using anti-FLAG or IgG antibodies and analyzed by LC-MS/MS (Fig. 6A). We identified seven viral proteins that were significantly enriched by CAD precipitation, and these proteins were UL19, UL21, UL42, UL31, UL47, UL37, and TK (Fig. 6A). To validate these interactions, HEK293T cells were co-transfected with plasmids expressing CAD and individual HSV-1 proteins, followed by co-immunoprecipitation assays. Interactions of CAD with UL19, UL21, UL42, UL31, UL47, and TK were successfully confirmed (Fig. 6B through H). The interaction between CAD and UL37 was relatively weak or transient to be reliably detected (Fig. 6G). Given that CAD deamidates RelA to suppress NF-κB activation, we next assessed how these CAD-binding proteins alter NF-κB activation using a luciferase reporter assay. Upon NF-κB activation by RelA overexpression, only UL42, UL47, and UL19 significantly reduced NF-κB activity among the seven CAD-binding HSV-1 proteins (Fig. 6I). To elucidate the mechanism of NF-κB inhibition, we examined RelA deamidation by two-dimensional gel electrophoresis (2-DGE). UL42 and UL47, but not UL19, shifted RelA toward the positive end of the gel strip, indicative of deamidation (Fig. S3A). It suggests that UL19 inhibits NF-κB activation through a RelA deamidation-independent mechanism. Notably, UL42 and UL47 did not further shift RelA-DD significantly toward the positive end, suggesting that these viral proteins primarily increase RelA deamidation at N64 and N139 (Fig. 6J). Consistent with UL42- and UL47-increased RelA deamidation, expression of UL42 or UL47 increased the heavy isotopologues of DHO in isotope-tracing experiments using [amide-^15^N]glutamine by three-fold (Fig. 6K). Neither viral protein altered the total abundance of DHO (Fig. S3B). This may be because UL42 and UL47 also promote the consumption of DHO for downstream pyrimidine synthesis. In contrast, UL19 failed to induce DHO synthesis and CAD activation. Together, these findings revealed that UL42 and UL47 play important roles in activating CAD during HSV-1 infection.

## DISCUSSION

Metabolic enzymes such as CAD are well characterized for catalyzing metabolite conversion, whereas our understanding of their functions beyond these biochemical reactions remains limited. In this study, we discovered that HSV-1 activates CAD to support pyrimidine synthesis and its lytic replication. However, uridine supplementation failed to fully restore HSV-1 replication in CAD-deleted cells, suggesting that CAD promotes HSV-1 replication through mechanisms beyond pyrimidine synthesis. Indeed, HSV-1 induces CAD activation to deamidate RelA, which effectively inhibits NF-κB-dependent gene expression while promoting glycolytic gene expression. RelA deamidation thus significantly suppresses host innate immune defense and fuels aerobic glycolysis to support biosynthesis underpinning HSV-1 lytic replication. In doing so, HSV-1 essentially couples the evasion of the antiviral inflammatory response to an activated cellular metabolic program to facilitate its productive infection and lytic replication. However, it is notable that CAD likely deamidates additional substrates besides RelA in HSV-1-infected cells, implying overlapping and distinct phenotypes in HSV-1 infection under the conditions of loss- or gain-of-function.

Immune response and cellular metabolism are two fundamental processes that compete with each other for the same pool of cellular resources. We have previously reported that CAD-mediated RelA deamidation occurs in a cell cycle-dependent manner, which peaks at S phase concomitant with the metabolic activity of CAD (26). Infectious pathogens, such as SARS-CoV-2 and HSV-1, tap the cellular mechanism to enable viral replication, suggesting the central role of CAD-mediated RelA deamidation in cell proliferation and metabolism. The featured roles of CAD-mediated RelA deamidation are shared by both HSV-1 and SARS-CoV-2, and potentially other viral pathogens, although the upstream activation may be distinct and specific for the viral pathogen. The SARS-CoV-2 Nsp9 protein acts as a bridge to induce CAD phosphorylation mediated by S6K1 (31). Interestingly, Nsp9 is part of the RNA replication machinery, raising the question of whether CAD activation by Nsp9 may contribute to viral RNA synthesis. Such a mechanism may choreograph an effective nucleotide channeling between nucleotide synthesis machinery and nucleotide polymerization enzyme complex.

In the present study, we identified two HSV-1 proteins, i.e., UL42 and UL47, which sufficiently activate CAD when ectopically expressed in cells. UL42 is defined as the DNA polymerase processivity factor that exponentially stimulates DNA polymerase activity in HSV-1 genome replication, thus possibly acting as a functional homolog of Nsp9 in SARS-CoV-2 biology (12, 13). Whether UL42-mediated CAD activation can directly influence DNA polymerization and viral genome replication remains unknown and will be further investigated. On the other hand, UL47 is one of the most abundant tegument proteins that are packaged into the virion (16). The tegument proteins are released into the cytoplasm of infected cells upon the membrane fusion between herpesvirus virions and host cells. Therefore, tegument-packaged UL47 and early expressed UL42 constitute a sequential mechanism that sustains CAD activation during immediate early and early phases of HSV-1 infection. Unfortunately, the molecular dissection of such a mechanism hinges on the identification of deletions of short sequences or point mutations within UL42 or UL47 that do not alter the key functions of UL42 and UL47, because both proteins are critical for efficient lytic replication of HSV-1. A prominent function of UL47 is its activity in RNA-binding and RNA metabolism, with the latter involving mRNA degradation mediated by the virion host shutoff (Vhs) RNAase (encoded by UL41) (18). This suggests that CAD may be involved in nucleotide metabolism in addition to nucleotide synthesis and RNA/DNA polymerization, further expanding the functional repertoire of CAD-mediated deamidation. Additionally, the molecular mechanisms by which these essential viral proteins activate CAD in HSV-1-infected cells remain to be elucidated. Interestingly, the Kaposi’s sarcoma-associated herpesvirus encodes vCyclin to activate CAD via a unique phosphorylation-mediated mechanism, hinting at the point that how CAD is regulated is not well understood (28). These are two main limitations of this study, which necessitate further experiments.

The fact that SARS-CoV-2 and HSV-1 of the RNA and DNA virus families, respectively, utilize CAD-mediated RelA deamidation to support viral replication not only supports the central role of this pathway but also alludes to a highly conserved mode of action of CAD in the infection of diverse viruses. Targeting CAD is expected to yield significant antiviral activity against these viral pathogens. Indeed, a small molecule inhibitor of CAD demonstrates potent antiviral effect in mouse models with SARS-CoV-2 infection (31). The effect of CAD inhibitors on HSV-1 and other DNA viruses remains to be explored. If successfully developed, these inhibitors may represent a new avenue to explore broad-spectrum antivirals targeting critical host factors. Such host-directed antivirals can act alone or in combination with virus-directed antivirals to overcome viral resistance, which is more pronounced for highly mutagenic viruses such as coronaviruses and influenza viruses. These CAD inhibitors may offer additional benefits than the better-defined inhibitors of dihydro-orotate dehydrogenase (DHODH) because of the deamidase and consequent regulatory roles beyond pyrimidine synthesis of CAD.

## MATERIALS AND METHODS

### Cell culture

HEK293T, Vero, human foreskin fibroblasts (HFFs), HCT116, and mouse embryonic fibroblasts (MEFs) were cultured in DMEM (HyClone) supplemented with 10% heat-inactivated FBS (Gibco) and 1% penicillin–streptomycin (Corning) and maintained in a humidified incubator with 5% CO_2_ at 37°C.

### Viral infection and titration

To perform HSV-1 infection, cells were infected with HSV-1 (strain 17) at indicated multiplicity of infection (MOI) in FBS-free DMEM medium for 1 h. Fresh DMEM medium containing 10% FBS was used to replace the infection medium after washing twice with PBS. The medium was collected at indicated time points post-infection for plaque assay to determine viral titer. The HSV-1 titer was determined by plaque assay on a Vero monolayer. In brief, a 10-fold serial-diluted virus-containing medium (FBS-free DMEM) was added to Vero cells. After 1 h of incubation in 5% CO_2_ at 37°C, DMEM containing 2% FBS and 1% methylcellulose (Sigma-Aldrich) was overlaid on infected Vero cells for plaque formation. At 3 days post-infection, Vero monolayers were stained with 1% crystal violet for plaque counting. All steps were performed in a Biosafety Level 2 lab.

### Lentivirus-mediated stable cell line construction

HEK293T cells were transfected with the packaging plasmids psPAX2 and pMD2.G and the pCDH lentiviral expression vector or lentiviral shRNA/sgRNA plasmids. At 48 h post-transfection, lentivirus-containing medium was harvested and filtered (and concentrated by ultracentrifugation if necessary). MEF and HFF were infected with the virus-containing medium in the presence of polybrene (8 μg/mL) with centrifugation at 1,800 rpm for 45 min. Cells were selected at 48 h post-infection and maintained in corresponding medium supplemented with puromycin (2 μg/mL) or hygromycin (200 μg/mL).

To generate reconstituted RelA-knockout (RelA-KO) MEFs with wild-type RelA, deamidated RelA-DD (containing N64D and N139D), or deamidation-resistant RelA-N64A, RelA-KO MEFs (from Chen et al. [32]) were infected with puromycin-resistant lentivirus containing wild-type, deamidated, or deamidation-resistant mutants of RelA. Reconstituted cells were then selected and maintained with puromycin, and protein expression was validated by immunoblotting.

HCT116 cells expressing RelA-WT or RelA-DD were generated in our previous publication (26). Briefly, to generate these cell lines, HCT116 cells were first infected with puromycin-resistant lentiviral shRNA targeting the 3′ UTR of RelA mRNA, and RelA depletion was validated by immunoblotting. RelA-depleted cells were then infected with hygromycin-resistant lentivirus expressing either wild-type RelA or RelA-DD. The reconstituted cells were selected and maintained with puromycin (1 μg/mL) and hygromycin (200 μg/mL). Expression of wild-type or mutant RelA-DD was validated using an anti-RelA antibody.

### Dual-luciferase reporter assay

HEK293T cells were transfected with an NF-κB reporter plasmid cocktail (100 ng of luciferase reporter plasmid and 10 ng of pRL Renilla luciferase control vector) and the expression plasmid by calcium phosphate precipitation. WCLs were used to determine the activity of firefly luciferase and Renilla luciferase by a microplate reader (FLUOstar Omega) at 24 h post-transfection.

### Quantitative real-time PCR (qRT-PCR)

Total RNA was extracted using TRIzol reagent (Invitrogen). Complementary cDNA was synthesized from total RNA using reverse transcriptase (Invitrogen). cDNA was diluted and analyzed by qRT-PCR using SYBR Green Master Mix (Applied Biosystems) with CFX Connect PCR instrument (Bio-Rad Laboratories). Relative mRNA expression for each target gene was calculated by the 2^−ΔΔCt^ method using β-actin as an internal control.

### Two-dimensional gel electrophoresis

Cells were lysed in rehydration buffer (7 M urea, 2 M thiourea, 2% CHAPS, 0.5% IPG buffer, and 0.002% bromophenol blue) by two pulses of sonication, and whole-cell lysates were centrifuged at 13,000 rpm for 15 min. Supernatants were loaded to IEF strips for focusing with a program comprising: 20 V, 10 h (rehydration); 500 V, 1 h; 1,000 V, 1 h; 1,000–5,000 V, 4 h; 5,000 V, 5 h. After IEF, strips were incubated with SDS equilibration buffer (50 mM Tris-HCl [pH 8.8], 6 M urea, 30% glycerol, 2% SDS, and 0.001% bromophenol blue) containing 10 mg/mL DTT for 15 min and then SDS equilibration buffer containing 2-iodoacetamide (25 mg/mL) for 15 min. Strips were washed with SDS-PAGE buffer, resolved by SDS-PAGE, and analyzed by immunoblotting.

### Co-immunoprecipitation (co-IP) and immunoblotting

HEK293T cells were transfected with the indicated expression plasmids for 48 h. Cells were harvested for experiments. Whole-cell lysates (WCLs) were prepared with NP40 buffer (50 mM Tris-HCl, pH 7.4, 150 mM NaCl, 1% NP-40, and 5 mM EDTA) supplemented with 20 mM β-glycerophosphate, 1 mM sodium orthovanadate, and a protease inhibitor cocktail (Roche). WCLs were sonicated and centrifuged, and the supernatant was pre-cleared with protein A/G agarose for 1 h. Pre-cleared samples were incubated with indicated antibodies overnight and protein A/G agarose for 1 h at 4°C. The agarose beads were washed extensively, and samples were eluted by boiling at 95°C for 10 min. Precipitated proteins and WCLs were analyzed by SDS gel electrophoresis and immunoblotting.

All immunoblotting was performed using the following primary antibodies: RelA (Invitrogen, PA5-16545, 1:2,000 dilution), CAD (Bethyl Laboratories, A301-374A, 1:2,000 dilution), Phospho-CAD (Ser1859) (Cell Signaling, 12662, 1:1,000 dilution), Flag (Sigma, F3165, 1:1,000 dilution), and V5 (Bethyl Laboratories, A190-120A, 1:1,000 dilution). Proteins were visualized by Odyssey infrared imaging system (LI-COR) after incubation with IRDye800-conjugated secondary antibodies (1:10,000 dilution, LI-COR).

### Metabolite profiling and isotope tracing

Roughly, half a million cells per sample (in triplicate) were harvested for metabolomics analysis. For isotope tracing experiments, cells were cultured with medium containing [U-^13^C]glucose (Cambridge Isotope Lab, cat. no. CLM-1396) or [^15^N-amide]glutamine (Cambridge Isotope Lab, cat. no. NLM-557-1). Metabolite extraction was performed after labeling. Cells were washed on ice with 1 mL ice-cold 150 mM ammonium acetate (NH_4_AcO, pH 7.3), and 1 mL of −80°C cold 80% MeOH was added to the wells. Samples were incubated at −80°C for 20 min, and cells were scraped off, and supernatant was transferred into microfuge tubes. Samples were pelleted at 4°C for 5 min at 15,000 rpm. The supernatant was transferred into new microfuge tubes. Metabolites were dried at room temperature under vacuum and re-suspended in water for LC-MS run.

Samples were randomized and analyzed on a Q-Exactive Plus hybrid quadrupole-Orbitrap mass spectrometer coupled to Vanquish UHPLC system (Thermo Fisher). The mass spectrometer was run in polarity-switching mode (+3.00 kV/−2.25 kV) with an *m/z* window ranging from 65 to 975. Mobile phase A was 5 mM NH_4_AcO, pH 9.9, and mobile phase B was acetonitrile. Metabolites were separated on a Luna 3 μm NH_2_ 100 NH_2_ 100 Å (150 × 2.0 mm) column (Phenomenex). The flow rate was 0.3 mL/min, and the gradient was from 15% A to 95% A in 18 min, followed by an isocratic step for 9 min and re-equilibration for 7 min. All samples were run in biological triplicate. Metabolites were detected and quantified as area under the curve based on retention time and accurate mass (5 ppm) using the TraceFinder 4.1 (Thermo Scientific) software. Raw data were corrected for natural isotope abundance and tracer impurity using the IsoCorrectoR package (33). All analyzed metabolic data are included as supplemental material (Table S2).

### Proteomics

To identify CAD-binding viral proteins, CAD and its binding proteins were precipitated with Anti-Flag or IgG agarose beads from HSV-1-infected 293T cells expressing Flag-CAD. After washing five times with 20 mM ammonium bicarbonate (pH 7.8), 10 mM DTT in 100 mM ammonium bicarbonate (pH 7.8) was added to the beads for reduction in a 37°C shaker for 30 min. Then, alkylation was performed by adding an equal volume of 25 mM IAA to the DTT bead suspension and rotating in the dark for 30 min at room temperature. Proteins were then digested by trypsin (1:100 enzyme/substrate ratio, 37°C shaker, overnight). Peptides were desalted with C18 Toptip and then dried in a rotary evaporator.

Purified peptides were re-suspended in 0.1% formic acid for LC-MS run. Briefly, peptides were separated with the C18 Acclaim PepMap column (75 μm id × 15 cm, 2 μm particle sizes, 100 Å pore sizes, Thermo Scientific) and ionized at 1.9 kV in positive ion mode. MS1 survey scans were acquired at a resolution of 70,000 from 350 to 1,800 *m/z*, with a maximum injection time of 100 ms and an AGC target of 1 × 10⁶. MS/MS fragmentation of the 14 most abundant ions was analyzed at a resolution of 17,500, AGC target 5e4, maximum injection time 65 ms, and normalized collision energy of 26. Dynamic exclusion was set to 20 s, and ions with a charge of +1, +7, and >+7 were excluded. MS/MS fragmentation spectra were searched with Proteome Discoverer SEQUEST (version 2.4, Thermo Scientific) against the *in silico* tryptic digested Uniprot all-reviewed Homo sapiens database (release June 2017, 42,140 entries). The maximum missed cleavages were set to 3. Dynamic modifications were set to oxidation on methionine (M, +15.995 Da) and deamidation on asparagine and glutamine (N and Q, +0.984 Da). Carbamidomethylation on cysteine residues (C, +57.021 Da) was set as a fixed modification. The maximum parental mass error was set to 10 ppm, and the MS/MS mass tolerance was set to 0.03 Da. The false discovery threshold was set strictly to 0.01 using the Percolator Node validated by *q* value. The relative abundance of parental peptides was calculated by integration of the area under the curve of the MS1 peaks using the Minora LFQ node. Spectral annotation was generated by the Interactive Peptide Spectral Annotator (IPSA, http://www.interactivepeptidespectralannotator.com/). Analyzed data are included as supplemental material (Table S1).

### Statistical analysis

Statistical analysis was performed using GraphPad Prism software for the two-way ANOVA test, one-way ANOVA test, or paired/unpaired two-tailed Student’s *t*-test. *P* values of <0.05 were considered statistically significant for each test.

## Data Availability

Raw proteomic data have been deposited in the ProteomeXchange Consortium via the PRIDE partner repository (Project accession: PXD079633). Metabolomic data are included as supplemental files. Any additional information reported in this paper is available from the lead contact upon request.

## SUPPLEMENTAL MATERIAL

Supplemental figures jvi.00920-26-s0001.pdf Fig. S1 to S3.

Table S1 jvi.00920-26-s0002.xlsx CAD-binding proteins in HSV-1-infected cells stably expressing CAD-Flag identified by affinity chromatography and tandem mass spectrometry.

Table S2 jvi.00920-26-s0003.xlsx Metabolite analysis in mock- or HSV-1-infected HFFs.

ASM does not own the copyrights to Supplemental Material that may be linked to, or accessed through, an article. The authors have granted ASM a non-exclusive, world-wide license to publish the Supplemental Material files. Please contact the corresponding author directly for reuse.
