# Supplementary material for: HSV-1 UL42 and UL47 hijack cellular nucleotide synthetic enzyme CAD to promote lytic replication
Source: J Virol. 2026 Sep 24;100(10):e00920-26. doi: 10.1128/jvi.00920-26 (PMC13712821; doi:10.1128/jvi.00920-26)
Supplement: Supplementary file 1 [file jvi.00920-26-s0001.pdf]

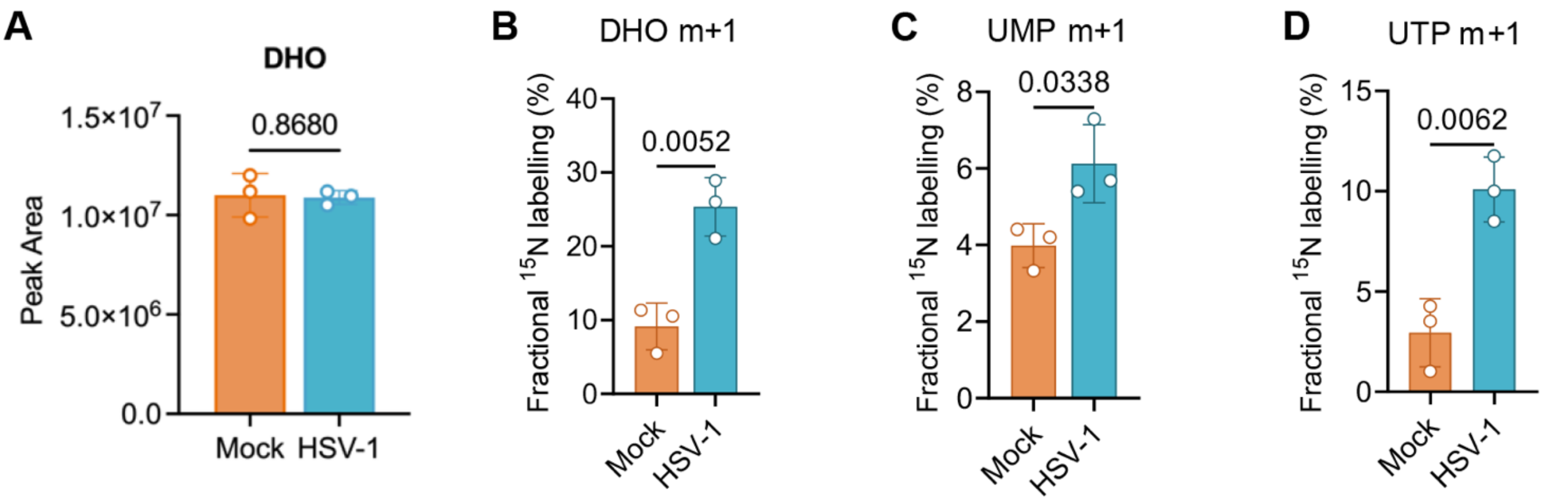

Figure S1. HSV-1 Activates CAD to Promote *de novo* Pyrimidine Synthesis

(A) DHO abundance in mock or HSV-1 infected MEFs (MOI=5, 6 h). (B-D) Fraction of the heavy isotopologues of indicated metabolites in mock or HSV-1 infected HFFs (MOI=5, 10 h) traced with [amide-^{15}N]glutamine for 15 minutes (for determining DHO labeling) or 1h (for determining UMP and UTP labeling). Data are presented as means ± SD of biological triplicates (A-D). Statistical analysis was performed by unpaired, two-tailed Student's t test (A-D).

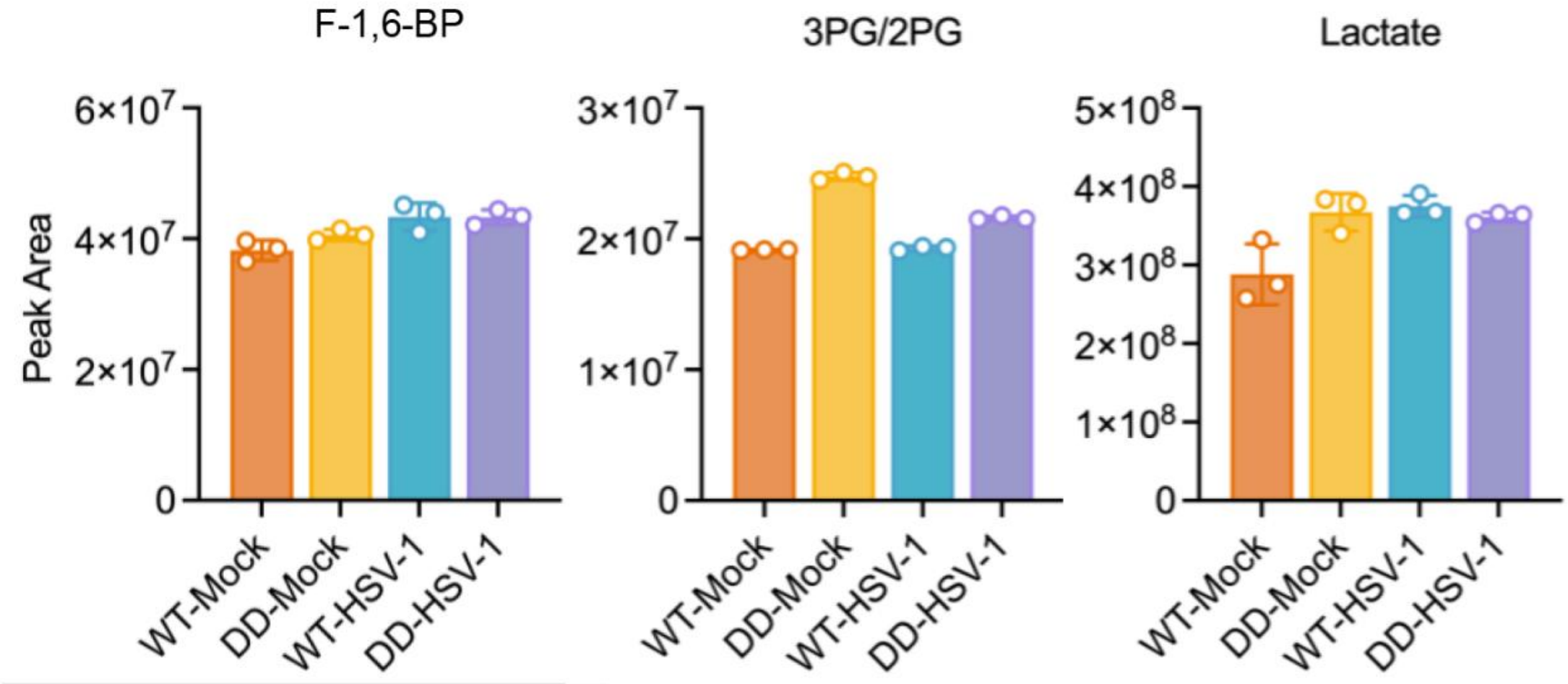

Figure S2. HSV-1 Promotes CAD-mediated RelA Deamidation to Upregulate Glycolysis
Abundance of indicated metabolites in RelA-KO MEFs reconstituted with wildtype or deamidated (DD) RelA infected with or without HSV-1 (MOI=5, 6 h). Data are presented as means ± SD of biological triplicates.

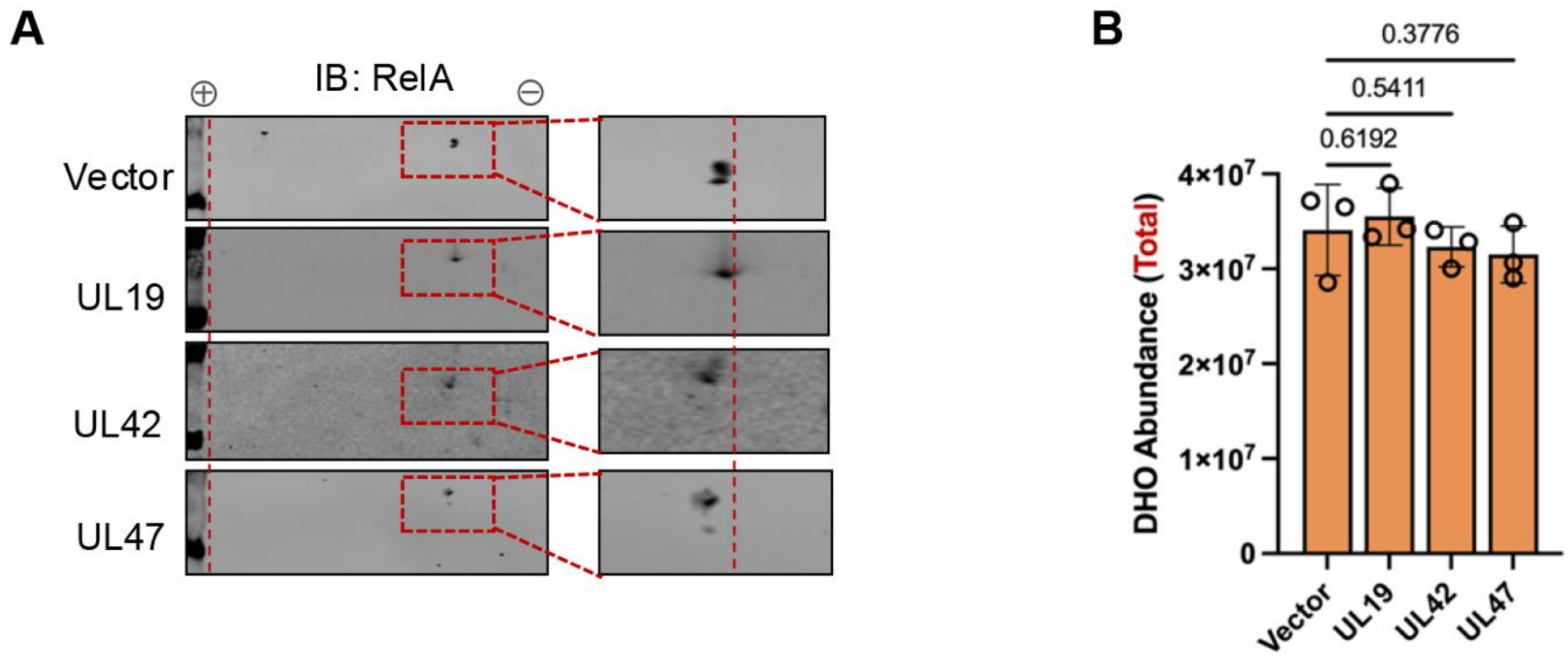

Figure S3. HSV-1 UL42 and UL47 Activate CAD.

(A) Immunoblots of whole cell lysates (WCLs) of HEK293T cells transfected with empty vector or plasmids expressing indicated viral proteins analyzed by two-dimensional gel electrophoresis (2DGE). (B) DHO abundance in HEK293T cells transfected with empty vector or plasmids expressing indicated viral proteins analyzed by LC-MS. Data are presented as means ± SD of biological triplicates (B) and are representative of three independent experiments (A). Statistical analysis was performed by the one-way ANOVA test (B).
